# Transcriptome analysis of iBET-151, a BET inhibitor alone and in combination with paclitaxel in gastric cancer cells

**DOI:** 10.5808/GI.2020.18.4.e37

**Published:** 2020-12-22

**Authors:** Sun Kyoung Kang, Hyun Joo Bae, Woo Sun Kwon, Jingmin Che, Tae Soo Kim, Hyun Cheol Chung, Sun Young Rha

**Affiliations:** 1 Songdang Institute for Cancer Research, Yonsei University College of Medicine, Seoul 03722, Korea; 2 MD Biolab Co., Ltd., Seoul 02455, Korea; 3 Brain Korea 21 PLUS Project for Medical Science, Yonsei University College of Medicine, Seoul 03722, Korea; 4 Division of Medical Oncology, Department of Internal Medicine, Yonsei Cancer Center, Yonsei University College of Medicine, Seoul 03722, Korea

**Keywords:** BET inhibitor, combination, differentially expressed genes, gastric cancer, paclitaxel, transcriptome

## Abstract

BET inhibitor, as an epigenetic regulator inhibitor, reduces the expression of oncogenes such as Myc and Bcl-2, which affects cancer growth and development. However, it has modest activity because of the narrow therapeutic index. Therefore, combination therapy is necessary to increase the anti-tumor effect. Paclitaxel, an anti-mitotic inhibitor, is used as second-line therapy for gastric cancer (GC) as a monotherapy or combination. In this study, we performed RNA sequencing of GC cells treated with iBET-151 and/or paclitaxel to identify the differentially expressed genes associated with possible mechanisms of synergistic effect. We also performed Gene Ontology enrichment and Kyoto Encyclopedia of Genes and Genomes pathway analyses to determine the most enriched terms and pathways of upregulated and downregulated genes. We found 460 genes in which iBET-151 and paclitaxel combination treatment changed more than single-treatment or no-treatment. Thus, additional functional studies are needed, but our results provide the first evidence of the synergistic effect between iBET-151 and paclitaxel in regulating the transcriptome of GC cells.

## Introduction

Gastric cancer (GC) is the second leading cause of cancer-related mortalities worldwide, especially in Asian countries. In South Korea, the incidence of GC was the highest in 2017 and GC ranked fourth among the causes of cancer-related deaths [1-4]. Despite advances in therapeutics, recurrence occurs in about half of the patients, and the prognosis of recurrent and metastatic GC is very poor [5-7]. Thus, new therapeutic strategies involving for tumor-specific molecular targets are critically needed to improve the outcome of recurrent and metastatic GC patients. Paclitaxel, which is one of the most commonly used standard chemotherapeutic drugs, binds to β-tubulin and stabilizes microtubules, resulting in G2/M arrest and cell death. Various targeted anticancer agents were tested in combination with paclitaxel as second-line therapy for advanced GC [8-11]. However, most combinations, except that with ramucirumab, failed to show improved efficacy [12]. Moreover, mechanisms underlying paclitaxel activity, except mitosis inhibition, have not yet been studied well. Elucidating these mechanisms is important for the selection of appropriate combination partners of paclitaxel.

Epigenetic dysregulation is a common feature of cancer, including GC. Eventually, it contributes to GC development and progression [13]. In our previous study, iBET-151, an inhibitor of the bromodomain and extraterminal (BET) protein, which is an epigenetic regulator, was found to function effectively in GC cells (unpublished data). A recent study reported that lowering the expression of B-cell lymphoma (BCL)-2 and BCL-xL, the targets of the BET protein, induces sensitivity to paclitaxel [14]. We confirmed that paclitaxel and iBET-151 exerted synergistic effects in GC cells by investigating cell viability and migration and the cell cycle (unpublished data). However, to determine the detailed synergistic mechanisms of paclitaxel and iBET-151, it is necessary to identify the overall changes in the transcriptome and associated pathways.

RNA sequencing (RNA-seq), via next-generation sequencing, is very useful for differential expression analysis involving specific conditions, such as ‘drug treatment versus no-treatment.’ Therefore, researchers can comprehensively report the gene expression changes related to drug treatment by performing RNA-based genome-wide profiling [15].

In this study, we performed RNA-seq to analyze the differentially expressed genes (DEGs) observed following treatment with paclitaxel, iBET-151, and their combination in GC cells. We also identified the biological and functional pathways associated with DEGs using the Gene Ontology (GO) and Kyoto Encyclopedia of Genes and Genomes (KEGG) databases to determine the possible action mechanisms of BET monotherapy and BET-paclitaxel combination treatment. 

## Methods

### Cell line and cell culture

AGS GC cell line (CRL-1739) was obtained from the American Type Culture Collection (ATCC, Manassas, VA, USA). AGS cells were cultured in Eagle's Minimum Essential Medium supplemented with 10% fetal bovine serum (Lonza, Basel, Switzerland) and 1× penicillin/streptomycin (Lonza). All experiments were performed after AGS cells reached the exponential growth phase.

### Drugs

A small-molecule inhibitor of the BET family, iBET-151, was purchased from Selleckchem (Houston, TX, USA) and prepared as a 10-mM stock solution in DMSO (Sigma-Aldrich, St. Louis, MO, USA). Paclitaxel, an anti-mitotic drug, was purchased from Sigma-Aldrich.

### RNA-seq data analysis

For differential expression analysis by RNA-seq, total RNA was isolated from iBET-151- or/and paclitaxel-treated or untreated AGS cells using the RNeasy Mini Kit (Qiagen, Hilden, Germany), according to the manufacturer's instructions. RNA quantity and integrity were assessed using both Nanodrop and Bioanalyzer, according to the manufacturer’s instructions. Library preparation and RNA-seq was performed using the Illumina TruSeq RNA Library Preparation Kit (Illumina, San Diego, CA, USA), according to the manufacturer’s protocol. Samples were sequenced on the Illumina NovaSeq 6000, 150 bp paired-end reads, to a minimum depth of 120 million reads per sample. Sequenced reads were aligned to the hg19 genome assembly using TopHat2. Differential expression analysis was processed following the Tuxedo protocol. DEGs were defined based on the cutoff values of p ≤ 0.05, false discovery rate < 0.05, and fold change (FC) > 1.5. The reference annotation based on Ensembl release 69 (ftp://ftp.ensembl.org/pub/release-69/gtf/macaca_mulatta).

### Functional enrichment analysis

GO annotation enrichment analysis was performed using The Database for Annotation, Visualization, and Integrated Discovery (DAVID) (v6.8), an online tool applied for functional annotation analysis and Gene Set Enrichment Analysis using MSigDB. GO and KEGG pathway analyses provide a comprehensive set of functional annotation tools for identifying characteristic biological attributes of high-throughput genome or transcriptome data.

### Western blot analysis

To study the effect of drugs on protein expression, tissues and cells were lysed in RIPA buffer (50 mM Tris [pH 8.0], 150 mM NaCl, 1% NP-40, 0.1% sodium dodecyl sulfate, 1 mM EDTA, 10% glycerol) containing protease and phosphatase inhibitors (Roche Diagnostics, Laval, QC, Canada). The cell lysates were subjected to sodium dodecyl sulfate polyacrylamide gel electrophoresis and western blotting. Membranes were then developed using an enhanced chemiluminescence (ECL) reagent (GE Healthcare, Piscataway, NJ, USA). α-tubulin was used as a loading control. Antibodies used for western blotting included anti‒epidermal growth factor receptor (EGFR), anti‒fibroblast growth factor receptor (FGFR)-3, and anti‒insulin-like growth factor-1 receptor (IGF-1R; Cell Signaling Technology, Beverly, MA, USA); anti‒human epidermal growth factor receptor (HER)-2, anti‒HER-3, and anti-MET (Santa Cruz Biotechnology, Santa Cruz, CA, USA); anti-α-tubulin (Sigma-Aldrich); and horseradish peroxidase-conjugated secondary anti-rabbit and anti-mouse (Jackson ImmunoResearch Laboratories, West Grove, PA, USA) antibodies. Membranes were then developed using the ECL reagent (GE Healthcare), and α-tubulin was used as a loading control. Quantification was performed with a Molecular Imager ChemiDoc XRS+ Imaging System (Bio-Rad, Hercules, CA, USA)

### Statistical analysis

Graphing was performed using GraphPad Prism software version 8 (Graph Pad Software Inc., San Diego, CA, USA). All statistical analyses were performed using SPSS version 25 (IBM Corp., Armonk, NY, USA). All the significance levels were set at p ≤ 0.05, and all p-values were two-sided.

## Results

### DEGs induced by iBET-151, paclitaxel, and combination treatments

In our previous study, we confirmed that iBET-151-paclitaxel combination treatment exerted a synergistic effect on cell viability and invasion in several cell lines (unpublished data). To understand the effects of iBET-151 and paclitaxel on the whole transcriptome better, we performed RNA-seq of AGS cells that were most affected by the synergistic effects of iBET-151 and paclitaxel to compare the global gene expression profiles of untreated and iBET-151-, paclitaxel-, and iBET-151‒paclitaxel combination-treated cells.

**Fig. 1.**
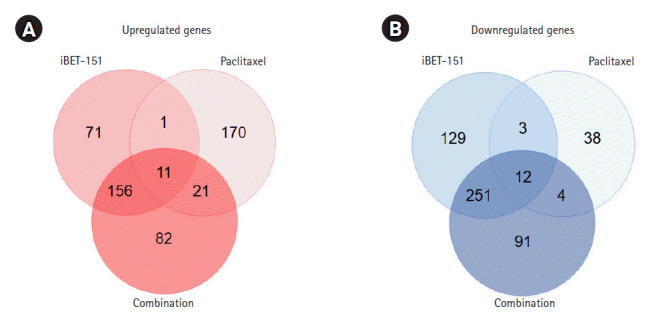
Overview of genes regulated by iBET-151, paclitaxel, and combination treatments. (A) Venn diagram analysis of the number of upregulated genes in treated versus untreated cells is shown. (B) Venn diagram analysis of the number of downregulated genes in treated versus untreated cells is shown.

About 16,000 genes were selected after the preprocessing step, during which genes that were not detected in both treated and untreated AGS cells were excluded. Based on our DEG-specific criteria, we observed that iBET-151 upregulated 239 genes, whereas paclitaxel upregulated 203 genes (Fig. 1A). Upregulation of 270 genes by combination treatment was mainly due to the effect of iBET-151 treatment (Fig. 1A). Of the genes upregulated by iBET-151 alone, 61% (167/270) were upregulated by the combination of iBET-151 and paclitaxel. Thus, these genes were shared between the combination- and iBET-151‒treated cells.

Then, we observed that iBET-151 downregulated 395 genes, whereas paclitaxel downregulated 57 genes (Fig. 1B). In the combination-treated cells, 358 genes were downregulated; among them, 73% (263/358) overlapped with the downregulated genes in the iBET-151‒treated cells (Fig. 1B). However, the genes regulated by paclitaxel alone did not overlap with those regulated by iBET-151 alone or the iBET-151-paclitaxel combination. The top 10 genes upregulated and downregulated by iBET-151, paclitaxel, and combination treatments are shown in Tables 1 and 2, respectively.

### GO functional enrichment analysis

**Fig. 2.**
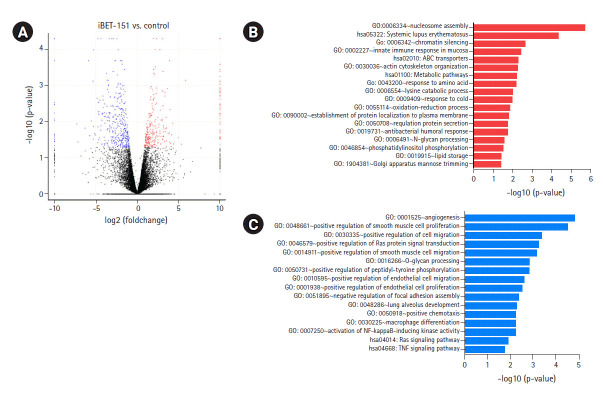
DEGs induced by iBET-151 treatment. (A) Volcano plot of DEGs induced in iBET-151‒treated versus untreated cells is shown. (B) GO biological processes/KEGG pathways upregulated in response to iBET-151 treatment are shown. (C) GO biological processes/KEGG pathways downregulated in response to iBET-151 treatment are shown. DEGs, differentially expressed genes; GO, Gene Ontology; KEGG, Kyoto Encyclopedia of Genes and Genomes.

**Fig. 3.**
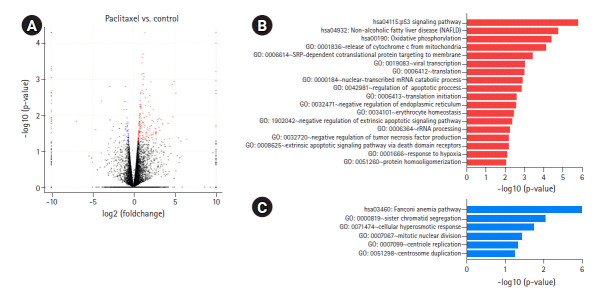
DEGs induced by paclitaxel treatment. (A) Volcano plot of DEGs induced in paclitaxel-treated versus untreated cells is shown. (B) GO biological processes/KEGG pathways upregulated in response to paclitaxel treatment are shown. (C) GO biological processes/KEGG pathways downregulated in response to paclitaxel treatment are shown. DEGs, differentially expressed genes; GO, Gene Ontology; KEGG, Kyoto Encyclopedia of Genes and Genomes.

**Fig. 4.**
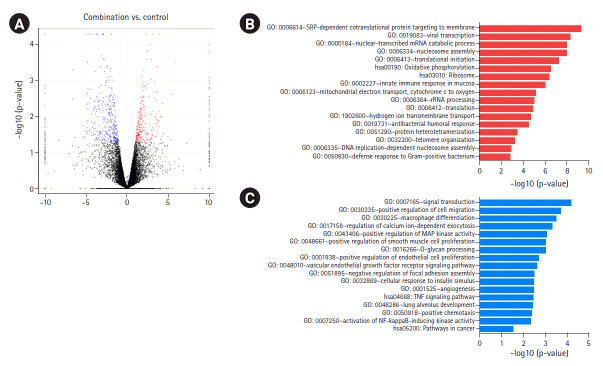
DEGs induced by iBET-151‒paclitaxel combination treatment. (A) Volcano plot of DEGs induced in combination-treated versus untreated cells is shown. (B) GO biological processes/KEGG pathways upregulated in response to iBET-151-paclitaxel combination treatment are shown. (C) GO biological processes/KEGG pathways downregulated in response to iBET-151-paclitaxel combination treatment are shown. DEGs, differentially expressed genes; GO, Gene Ontology; KEGG, Kyoto Encyclopedia of Genes and Genomes.

To determine the biological effects of gene expression changes, we performed GO analysis with the selected DEGs using DAVID v6.8. Volcano plots were used for the concurrent demonstration of both FC and p-value of DEGs in mono- or combination-treated cells (Figs. 2A, 3A, and 4A).

Interestingly, GO analysis showed that upregulated genes in iBET-151‒treated cells were most enriched in the chromatin silencing, nucleosome assembly pathways, and DNA packing complex (Fig. 2B, Supplementary Fig. 1A). This was because iBET-151 suppressed the epigenetic regulatory function of the BET family and affected chromatin remodeling. The genes downregulated by iBET-151 were enriched in angiogenesis; cell migration; RAS protein signal transduction; positive regulation of extracellular signal-regulated kinase (ERK)-1 and ERK cascade; positive regulation of transcription, DNA-templated; and negative regulation of apoptotic process (Fig. 2C, Supplementary Fig. 1B).

In paclitaxel-treated cells, the upregulated genes were enriched in the apoptotic signaling pathway; release of cytochrome c from mitochondria; regulation of apoptotic process; and mitochondrial electron transport, cytochrome c to oxygen (Fig. 3B, Supplementary Fig. 1C). Genes downregulated by paclitaxel were enriched in sister chromatid segregation, mitotic nuclear division, and centrosome duplication (Fig. 3C, Supplementary Fig. 1D). According to a recent study [16], paclitaxel inhibited mitosis by binding to microtubules and regulating mitosis-related pathways, including mitotic nuclear division, chromosome segregation, and G2/M transition of the mitotic cell cycle, in lung cancer cells. The results of our previous study on lung cancer cells showed that paclitaxel upregulated mitosis-related pathways, including mitotic nuclear division and chromosome segregation (unpublished data).

GO analysis showed a significant increase in the upregulation of the same genes involved in the aforementioned pathways in combination-treated cells. Moreover, an increase in the upregulation of genes involved in reactive oxygen species metabolic process and nucleotide-excision repair, DNA damage recognition was observed after combination treatment (Fig. 4B, Supplementary Fig. 1E). We observed that downregulated genes in the combination- and iBET-151-treated cells were enriched in overlapping GO terms, including angiogenesis, positive regulation of RAS protein signaling, cell migration, MAP kinase activity, and positive regulation of signaling; however, a significant decrease in gene expression was observed between the combination- and mono-treated cells (Fig. 4C, Supplementary Fig. 1F).

### Significantly altered genes in combination-treated cells, compared with that in mono-treated cells

To understand the effects of combination treatment better, we identified genes that were more significantly upregulated or downregulated in combination-treated cells than in mono-treated cells. We first identified the genes with FC > 1.5 and p < 0.05 in combination-treated cells, compared with in control cells. We then identified the DEGs with FC > 1.5 in the respective mono-treated cells.

**Fig. 5.**
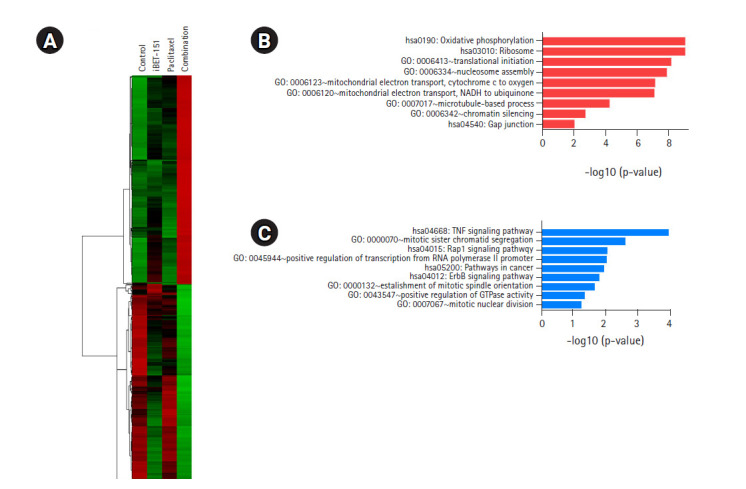
DEGs induced by iBET-151‒paclitaxel combination treatment. (A) Volcano plot of DEGs induced in combination-treated versus untreated cells is shown. (B) GO biological processes/KEGG pathways upregulated in response to iBET-151-paclitaxel combination treatment are shown. (C) GO biological processes/KEGG pathways downregulated in response to iBET-151-paclitaxel combination treatment are shown. DEGs, differentially expressed genes; GO, Gene Ontology; KEGG, Kyoto Encyclopedia of Genes and Genomes.

We identified 460 DEGs, including 238 upregulated and 222 downregulated DEGs, in AGS cells based on the above-mentioned cutoff values. As shown in Fig. 5A, the heatmap of hierarchical clustering of 460 DEGs demonstrated clear discriminating patterns. Then, GO analysis demonstrated that combination treatment upregulated genes enriched in nucleosome assembly, mitochondrial electron transport, and microtubule-based process (Fig. 5B). Subsequent GO analysis after combination treatment showed downregulation of genes involved in mitotic sister chromatid segregation, positive regulation of transcription, positive regulation of GTPase activity, and mitotic nuclear division (Fig. 5C).

Most of these results after combination treatment were similar to those obtained after iBET-151 or paclitaxel mono-treatment. However, combination treatment induced more changes in the genes than those in case of either of the mono-treatments.

### Expression of BET protein target genes and major receptor tyrosine kinase

**Fig. 6.**
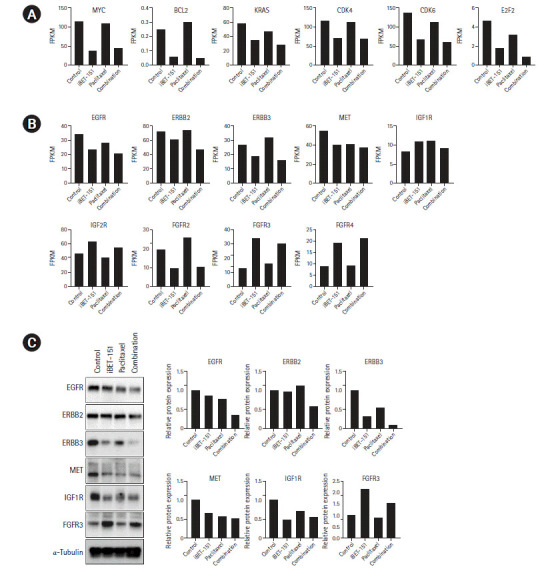
Expression of BET protein target genes and major RTK. (A) mRNA expression of BET family protein target and tumor growth-related genes is shown. (B) GO biological processes upregulated in response to combination treatment are shown. (C) Validation of DEGs was performed by western blotting. BET, bromodomain and extraterminal; RTK, receptor tyrosine kinase; GO, Gene Ontology; DEGs, differentially expressed genes; FPKM, fragments per kilobase of exon model per million reads mapped; EGFR, epidermal growth factor receptor; IGF1R, insulin-like growth factor receptor 1; IGF2R, insulin-like growth factor receptor 2; FGFR, ﬁbroblast growth factor receptor.

Next, we determined the mRNA expression levels of BET protein target genes. We observed that iBET-151 reduced the mRNA expression of the oncogenes, MYC and KRAS, and the anti-apoptotic gene, BCL-2. However, no change was observed in the expression of these genes on paclitaxel treatment, as expected (Fig. 6A).

In our previous study, we found that iBET-151 induced G1 cell cycle arrest (unpublished data). Consistent with this result, the expression of cyclin dependent kinase-4/6 and E2F transcription factor-2 involved in G1-S phase transition was reduced. To elucidate the underlying biology, we observed the changes in major receptor tyrosine kinase (RTK) expression. We confirmed that iBET-151 reduced the expression of RTK mRNA and proteins, including EGFR, ERBB3, MET, and IGF-1R, thereby regulating cancer cell growth and survival. Additionally, RTK expression was further reduced on iBET-151‒paclitaxel combination treatment. However, in the FGFR family, FGFR-3/4 was significantly increased on iBET-151 and combination treatments (Fig. 6B and 6C).

## Discussion

In this study, we performed RNA-seq to identify the transcriptome changes induced by mono- and combined treatments with iBET-151 and paclitaxel in the AGS GC cell line. First, we confirmed that the expression of MYC and BCL-2, which is known as the target of BRD-4, was decreased on iBET-151 treatment, as expected. Interestingly, we observed that KRAS, an oncogene that plays a key role in the development and progression of various carcinomas, was downregulated in iBET-151-treated cells. Even though KRAS has been known as a significant driver in various cancers, it has long been considered undruggable. Interestingly, we confirmed that KRAS mRNA expression was reduced on iBET-151 treatment and further decreased on iBET-151‒paclitaxel combination treatment. This result suggested that iBET-151 could be developed as an indirect KRAS inhibitor for potential therapeutic effects in GC patients.

Additionally, we confirmed that expression of signal transduction-related genes, such as epidermal growth factor, vascular endothelial growth factor, IGF, and SRC, was also significantly decreased. On the other hand, expression of genes related to chromatin silencing and nucleosome assembly was increased on iBET-151 treatment. This was because the BET family proteins, the targets of iBET-151, are epigenetic regulators. Particularly, expression of sirtuin (SIRT)-4 involved in histone deacetylation was significantly increased. SIRT-4 is a tumor suppressor that is reduced in various carcinomas, and reduced SIRT-4 protein expression is associated with poor prognosis. It has also been reported that SIRT-4 interferes with glutamine metabolism and inhibits cell proliferation and migration [17]. As mentioned in our previous study, the metabolism pathway is enhanced in not only the iBET-151‒sensitive group, but also various carcinomas (unpublished data). Therefore, we expected that increasing SIRT-4‒inhibiting glucose pyruvate dehydrogenase using iBET-151 would be a feasible therapeutic strategy for these cancers.

Paclitaxel is a drug that binds to tubulin and stabilizes microtubules to suppress mitosis. However, our RNA-seq results revealed that paclitaxel also regulates expression of genes involved in cell division in GC. In this study, expression of genes related to sister chromatid segregation, mitotic nuclear division, and centrosome duplication was decreased in paclitaxel-treated GC cells. Additionally, expression of genes involved in cytochrome c release in mitochondria and apoptotic process was increased. This suggested that paclitaxel not only inhibited mitosis by binding to tubulin but also regulated mitosis inhibition and apoptosis in cell death by regulating related gene expression. Therefore, our study provides novel findings that may help in identifying appropriate combination drugs with paclitaxel for GC treatment via determination of gene expression changes induced by paclitaxel.

Additionally, we found that iBET-151 and paclitaxel separately reduced the expression of RTK, which is involved in key signaling pathways (RAS/mitogen-activated protein kinase and phosphoinositide 3-kinase/AKT pathways) that regulate cancer cell proliferation.

Trastuzumab is approved for use as HER-2‒targeted therapy for various cancers, including GC, to improve survival of patients with HER-2 amplification. However, in many cases, acquired resistance is induced by re-activation due to interaction with other RTKs, and this is related to poor survival. We found that iBET-151 reduced the expression of other RTKs, such as EGFR, HER-3, and MET, which are induce signaling activation by heterodimeric binding to HER-2. This expression is further decreased on iBET-151-paclitaxel combination treatment. Therefore, the combination of iBET-151 and paclitaxel can overcome resistance induced by HER-2-targeted treatment in GC.

JQ1, a BET inhibitor, has been recently reported to induce resistance by increasing FGFR protein expression in ovarian cancer [18] and uveal melanoma [19], but the underlying mechanism remains unclear. In our results, FGFR-3 protein expression was increased in GC, and RNA-seq showed that FGFR-3 and FGFR-4 mRNA expression was increased. Further research is needed to elucidate FGFR-3 regulation, but this makes it possible to find out that FGFR3 is regulated at the transcription level by the BET inhibitor. Therefore, our results show that not only JQ1 but also iBET-151 increased FGFR-3 expression, and in particular iBET-151 regulates FGFR3 at the mRNA level. Therefore, our results add evidence for combination therapy with BET inhibitor and FGFR inhibitor.

In our previous study, iBET-151 and paclitaxel exerted synergistic effects on GC cell proliferation and migration (unpublished data). Using RNA-seq analysis, we found that combination-treated cells showed higher changes in gene expression in the above-mentioned pathways than untreated and mono-treated cells. AGS cells showed the greatest reduction in angiogenesis-related expression of vascular endothelial growth factor-A, matrix metalloproteinase-14, and X-box-binding protein (XBP)-1 in response to combination treatment, compared with to either iBET-151 or paclitaxel single treatment. Particularly, XBP-1, a transcription factor, is known to promote not only angiogenesis, but also tumorigenesis, proliferation, and drug resistance.

In conclusion, we analyzed the biological functions induced by iBET-151-paclitaxel combination treatment. The results elucidated the biological mechanism underlying the synergistic effects of iBET-151 and paclitaxel. More research is needed in the future to determine the exact mechanisms underlying the action of these compounds, but we expect that the combination therapy of iBET-151 and paclitaxel will be a promising therapeutic strategy for GC.

## Figures and Tables

**Table 1.** Top 10 upregulated genes in AGS cells treated by iBET-151 and paclitaxel

Gene name	Fold change	p-value	Function
iBET-151			
*EFR3B*	5.880	0.0007	Membrane-anchoring component
*GPR157*	5.380	0.0415	Cell surface receptor signaling pathway, G-protein coupled receptor signaling pathway
*STK31*	4.980	0.0272	RNA catabolic process
*IGFN1*	4.440	0.0409	Cytokine-mediated signaling pathway, response to lipopolysaccharide, regulation of synapse organization
*IL10RA*	4.330	0.0111	Cytokine-mediated signaling pathway, response to lipopolysaccharide, regulation of synapse organization
*CLUL1*	4.180	0.0228	Cell death
*SPOCK2*	4.140	0.0484	Positive regulation of cell-substrate adhesion
*TEX14*	4.080	0.0392	Mitotic spindle assembly checkpoint
*HIST2H4A*	3.980	0.0046	Chromatin silencing at rDNA, negative regulation of gene expression (epigenetic)
*CLU*	3.920	0.0002	Release of cytochrome c from mitochondria, intrinsic apoptotic signaling pathway
Paclitaxel			
*BMP7*	86.2229	0.0465	Secreted ligand of the TGF-beta
*KCNQ5*	43.4113	0.04195	Regulating potassium channel
*AMPD3*	34.5353	0.00245	Catalyzes the deamination of AMP to IMP in red cells and plays an important role in the purine nucleotide cycle.
*TSPAN32*	29.6508	0.00115	Functional roles in cell motility, membrane fusion, proliferation, and adaptive immunity
*MAGED1*	27.0958	0.0111	Inhibits cell cycle progression, and facilitates NGFR-mediated apoptosis
*MYO15A*	25.4572	0.0014	Actin-based motor molecules with ATPase activity
*ZDHHC1*	20.3930	0.04455	Innate immune response, related to phencyclidine abuse
*CATSPERE*	18.0009	0.01695	Calcium channel pore-forming proteins
*ADCY10P1*	12.9960	0.0478	Sphingosine metabolic process
*MYLK*	11.4716	0.0186	Activated by the binding of calcium-calmodulin, interaction with actin filaments to produce contractile activity
Combination			
*EFR3B*	55.7152	0.0003	Acts as the membrane-anchoring component
*MMP1*	42.8137	0.0024	Calcium ion binding and metallopeptidase activity
*HIST1H2BJ*	32.0000	0.0362	Wrap and compact DNA into chromatin, limiting DNA accessibility to the cellular machineries which require DNA as a template
*SELENOM*	22.0087	0.02955	Function as a thiol-disulfide oxidoreductase that participates in disulfide bond formation
*HIST2H4A*	20.2521	0.0042	Wrap and compact DNA into chromatin, limiting DNA accessibility to the cellular machineries which require DNA as a template
*TEX14*	19.0273	0.035	Kinetochore-microtubule attachment during mitosis
*HIST1H2BC*	17.5087	0.04265	Wrap and compact DNA into chromatin, limiting DNA accessibility to the cellular machineries which require DNA as a template
*HIST1H2BG*	17.2677	0.0098	Wrap and compact DNA into chromatin, limiting DNA accessibility to the cellular machineries which require DNA as a template

**Table 2.** Top 10 downregulated genes in AGS cells treated by iBET-151 and paclitaxel

Gene name	Fold change	p-value	Function
iBET-151			
*ADGRF1*	0.006	0.0116	Cell surface receptor signaling pathway, G-protein coupled receptor signaling pathway
*PMEPA1*	0.007	0.0362	Cell proliferation, differentiation, apoptosis, motility, extracellular matrix production and immunosuppression
*PSG1*	0.013	0.015	Defense response
*NTM*	0.017	0.0002	Neural cell adhesion molecule, cell adhesion
*CACNA1H*	0.019	0.02405	Regulation of ion transmembrane transport, calcium ion transmembrane transport
*SLAMF7*	0.021	0.00945	Adaptive immune response, cell adhesion, natural killer cell activation
*BMP4*	0.025	0.00075	Activation of MAPKK activity
*NRP2*	0.044	0.00255	Angiogenesis, positive regulation of endothelial cell proliferation
*TCF4*	0.048	0.00005	Activate transcription
*SRC*	0.136	0.00005	Epidermal growth factor receptor signaling pathway, signal transduction
Paclitaxel			
*DOCK10*	0.008	0.0147	Regulation of CDC42 activity
*ADAP2*	0.042	0.0253	GTPase activator activity
*SYT1*	0.067	0.00405	Calcium ion binding and transporter activity
*NREP*	0.139	0.0016	Neural function
*MEGF11*	0.14	0.0136	Retina layer formation, motypic cell-cell adhesion
*FAM19A2*	0.149	0.04425	Neuronal survival and neurobiological functions
*GCNT2*	0.168	0.02685	Acetylglucosaminyltransferase activity and N-acetyllactosaminide beta-1,6-N-acetylglucosaminyltransferase activity
*TTYH1*	0.187	0.02065	Cell adhesion
*RMDN2*	0.308	0.0235	Interact with microtubules
*NCKAP5*	0.31	0.0394	Microtubule depolymerization
Combination			
*GBP1*	0.003	0.04585	GTPase activity
*INHBE*	0.011	0.0005	Insulin secretion, nerve cell survival, cytokine-cytokine receptor interaction
*NUPR1*	0.012	0.03795	Positively regulates cell cycle progression
*GPX5*	0.024	0.0382	Protects cells and enzymes from oxidative damage
*DOCK10*	0.024	0.01775	Regulation of CDC42 activity
*RP1*	0.028	0.03635	Microtubule-associated protein regulating the stability and length of the microtubule-based axoneme of photoreceptors
*PMEPA1*	0.029	0.0249	A negative regulator of TGF-beta signaling
*GCNT3*	0.033	0.0025	Synthesize all known mucin beta 6 N-acetylglucosaminides
*SLAMF7*	0.034	0.006	Regulation and interconnection of both innate and adaptive immune response
*PHF21B*	0.034	0.0201	Metal ion binding

## Supplementary Materials

Supplementary Fig 1. DEGs induced by iBET-151, paclitaxel, and combination treatments. (A) Enriched GO/KEGG gene sets identified using GSEA for genes upregulated in AGS cells in response to iBET-151 vs. Control. (B) Enriched GO/KEGG gene sets identified using GSEA for genes downregulated in AGS cells in response to iBET-151 vs. control. (C) Enriched GO/KEGG gene sets identified using GSEA for genes upregulated in AGS cells in response to paclitaxel vs. control. (D) Enriched GO/KEGG gene sets identified using GSEA for genes downregulated in AGS cells in response to paclitaxel vs. control. (E) Enriched GO/KEGG gene sets identified using GSEA for genes upregulated in AGS cells in response to iBET-151 and paclitaxel in combination. (F) Enriched GO/KEGG gene sets identified using GSEA for genes downregulated in AGS cells in response to iBET-151 and paclitaxel in combination. DEGs, differentially expressed genes; GO, Gene Ontology; KEGG, Kyoto Encyclopedia of Genes and Genomes; GSEA, Gene Set Enrichment Analysis.
