# Supplementary material for: Transcriptome analysis of iBET-151, a BET inhibitor alone and in combination with paclitaxel in gastric cancer cells
Source: Genomics Inform. 2020 Dec 22;18(4):e37. doi: 10.5808/GI.2020.18.4.e37 (PMC7808866; doi:10.5808/GI.2020.18.4.e37)
Supplement: Supplementary Fig 1. — DEGs induced by iBET-151, paclitaxel, and combination treatments. (A) Enriched GO/KEGG gene sets identified using GSEA for genes upregulated in AGS cells in response to iBET-151 vs. Control. (B) Enriched GO/KEGG gene sets identified using GSEA for genes downregulated in AGS cells in response to iBET-151 vs. control. (C) Enriched GO/KEGG gene sets identified using GSEA for genes upregulated in AGS cells in response to paclitaxel vs. control. (D) Enriched GO/KEGG gene sets identified using GSEA for genes downregulated in AGS cells in response to paclitaxel vs. control. (E) Enriched GO/KEGG gene sets identified using GSEA for genes upregulated in AGS cells in response to iBET-151 and paclitaxel in combination. (F) Enriched GO/KEGG gene sets identified using GSEA for genes downregulated in AGS cells in response to iBET-151 and paclitaxel in combination. DEGs, differentially expressed genes; GO, Gene Ontology; KEGG, Kyoto Encyclopedia of Genes and Genomes; GSEA, Gene Set Enrichment Analysis. [file gi-2020-18-4-e37-suppl1.docx]

**Supplementary Figure 1.**


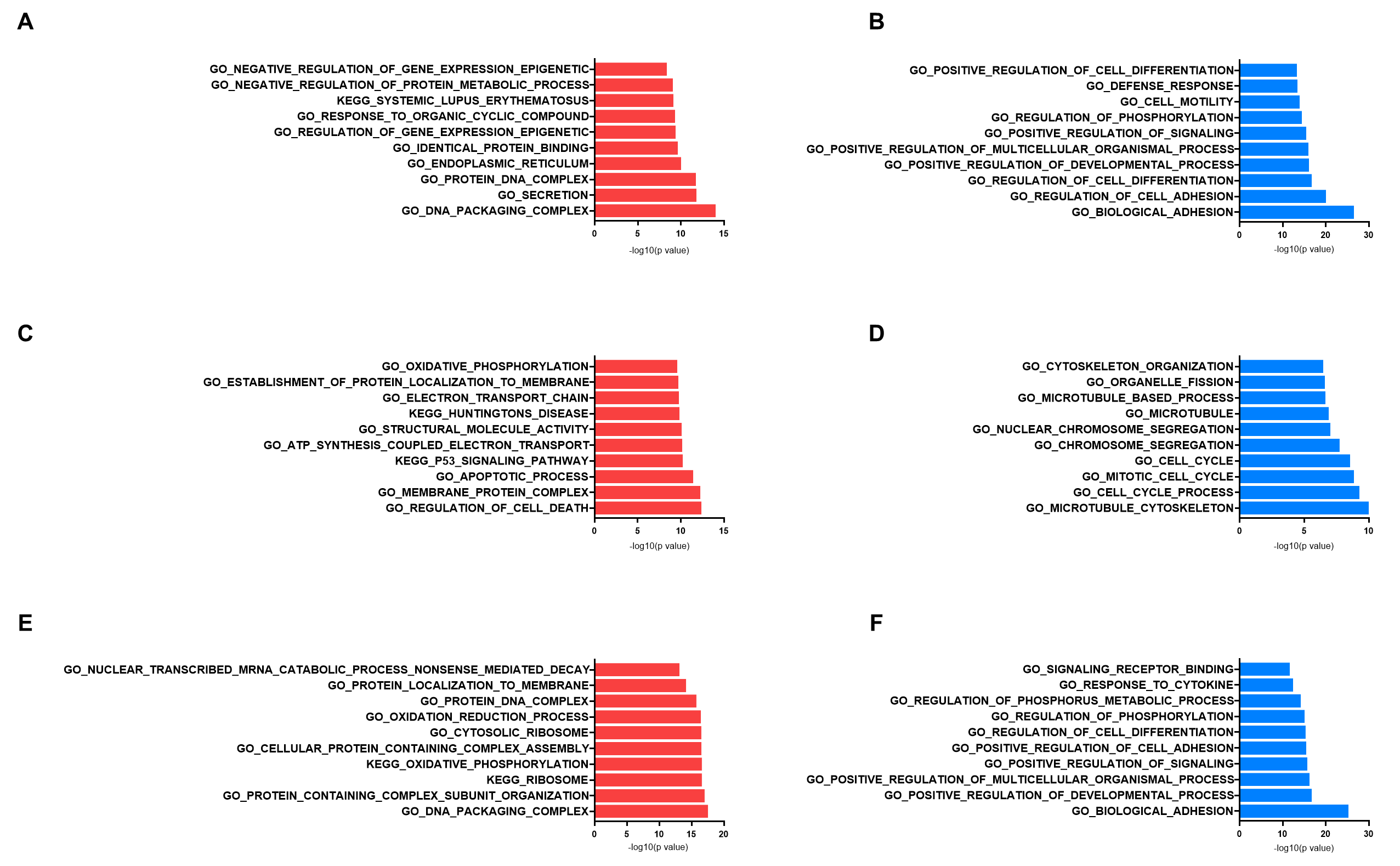


**Supplementary Fig. 1.** DEGs induced by iBET-151, paclitaxel, and combination treatments. (A) Enriched GO/KEGG gene sets identified using GSEA for genes upregulated in AGS cells in response to iBET-151 vs. Control. (B) Enriched GO/KEGG gene sets identified using GSEA for genes downregulated in AGS cells in response to iBET-151 vs. control. (C) Enriched GO/KEGG gene sets identified using GSEA for genes upregulated in AGS cells in response to paclitaxel vs. control. (D) Enriched GO/KEGG gene sets identified using GSEA for genes downregulated in AGS cells in response to paclitaxel vs. control. (E) Enriched GO/KEGG gene sets identified using GSEA for genes upregulated in AGS cells in response to iBET-151 and paclitaxel in combination. (F) Enriched GO/KEGG gene sets identified using GSEA for genes downregulated in AGS cells in response to iBET-151 and paclitaxel in combination. DEGs, differentially expressed genes; GO, Gene Ontology; KEGG, Kyoto Encyclopedia of Genes and Genomes; GSEA, Gene Set Enrichment Analysis.
